# Chemical chaperones reduce ER stress and adipose tissue inflammation in high fat diet-induced mouse model of obesity

**DOI:** 10.1038/srep27486

**Published:** 2016-06-08

**Authors:** Yaqin Chen, Zhihong Wu, Shuiping Zhao, Rong Xiang

**Affiliations:** 1 Department of Cardiology, the Second Xiangya Hospital of Central South University, Changsha, 410011, China; 2 The State Key Laboratory of Medical Genetics & School of Life Sciences, Central South University, Changsha, 410013, China

## Abstract

Obesity, which is characteristic by chronic inflammation, is defined as abnormal or excessive fat accumulation in adipose tissues. Endoplasmic reticulum (ER) stress is increased in adipose tissue of obese state and is known to be strongly associated with chronic inflammation. The aim of this study was to investigate the effect of ER stress on adipokine secretion in obese mice and explore the potential mechanisms. In this study, we found high-fat diet induced-obesity contributed to strengthened ER stress and triggered chronic inflammation in adipose tissue. Chemical chaperones, 4-PBA and TUDCA, modified metabolic disorders and decreased the levels of inflammatory cytokines in obese mice fed a high-fat diet. The alleviation of ER stress is in accordance with the decrease of free cholesterol in adipose tissue. Furthermore chemical chaperones suppress NF-κB activity in adipose tissue of obese mice *in vivo*. *In vitro* studies showed IKK/NF-κB may be involved in the signal transduction of adipokine secretion dysfunction induced by ER stress. The present study revealed the possibility that inhibition of ER stress may be a novel drug target for metabolic abnormalities associated with obesity. Further studies are now needed to characterize the initial incentive of sustained ER stress in obese.

Obesity is characterized by chronic low-grade inflammation, abnormal adipokines production and insulin resistance^1^,^2^,^3^. Secretory profile of adipocytes in obesity is shifted towards the proinflammatory, atherogenic and diabetogenic pattern. The expression and secretion of protective adipokines by adipose tissue such as adiponectin decreased while inflammatory cytokines elevated in obese state^4^. Altered adipokine secretion may represent a link between adipose tissue dysfunction in obesity and obesity-related metabolic diseases^5^. Although the features of chronic inflammation in obese adipose tissue are clearly defined, the signals and mechanism that trigger chronic inflammation is not well understood.

Newly synthesized secretory and membrane-associated proteins are correctly folded and assembled by chaperones in the endoplasmic reticulum (ER)^6^. The ER of adipocytes plays a major role in the assembly and secretion of adipokines. Failure of the ER’s adaptive capacity results in activation of the ER stress, also known as unfolded protein response (UPR). Recent studies have reported that ER stress is increased in adipose tissue of obese mice and human subjects^7^,^8^. Recently *in vitro* and *in vivo* evidence demonstrate a strong and causal relation between the functional capacity of ER and chronic inflammation in adipose tissue^9^, suggesting the possibility of exploiting this mechanism for therapeutic application.

Chemical or pharmaceutical chaperones, such as 4-phenyl butyric acid (4-PBA), ursodeoxycholic acid and its taurine-conjugated derivative (TUDCA), are a group of low molecular weight compounds known to stabilize protein conformation in the ER, and facilitate the trafficking of mutant proteins^10^. It can repress ER stress/UPR activation *in vitro* and *in vivo*^11^,^12^. Theoretically, the action of putative chemical chaperones may alleviates adipose tissue inflammation in obesity by inhibit ER stress, and thereby modify metabolic dysfunction.

ER stress and the UPR are linked to major inflammatory and stress-signaling networks via several distinct mechanisms, including the activation of IKB kinase nuclear factor κB (IKK-NF-κB) and JNK-AP-1 pathways. NF-κB plays an important role in adipocytes inflammation in human and experimental models of metabolic disease^13^ and is the main transcription factor in ER stress-mediated inflammation^14^. However, the signaling pathway between ER stress and chronic low-grade inflammation in adipose tissue in obesity is not very clear.

The aim of the present study was to investigate whether chemical chaperones, 4-PBA and TUDCA, can improve adipose inflammation in diet-induced obesity mice model by depressing ER stress. In addition, we studied whether the inflammation signaling pathway IKK-NF-κB participate in proinflammatory adipokines production induced by ER stress.

## Materials and Methods

### Cell culture and treatment

3T3-L1 preadipocytes were cultured and induced to differentiate into mature adipocytes as described previously^15^. Differentiated adipocytes were serum starved for 18 hours in DMEM supplemented with 0.2% bovine serum albumin (BSA) before treatment. Tunicamycin(TM) causes ER stress by inhibiting N-linked glycosylation. For the experiment, adipocytes were exposed to PS1145 (a potent inhibitor of NF-κB, 10 μM) for 24 h, followed by treatment with 0.6 mg/ml TM for 12 h.

### Mouse studies

5-week-old male C57BL/6 mice were housed under a 12-h light/12-h dark cycle, and followed free access to a high-fat diet (34.9%; D12492; Research Diets Inc.) to induce obesity or regular diet (RD, 6% fat; Oriental Yeast). After 14 weeks on the diet, mice were orally administered with 4-PBA or TUDCA twice daily in two divided doses (500 mg/kg at 8:00 am and 8:00 pm; total 1 g/kg/day) for 6 weeks. Mice in the control groups received the same volume of vehicle by oral gavage. The experimental procedures and housing conditions were approved by the Committee of Animal Experimentation, The Centre South University. All experiments were performed in accordance with relevant guidelines and regulations.

### Western blotting

Cell lysates were prepared with RIPA lysis solution (Beyotime Institute of Biotechnology, China). Protein concentration was determined using the bicinchoninic acid (BCA) protein assay kit (Pierce).Equivalent amounts of protein were denatured and subjected to 15% sodium dodecyl sulfate-polyacrylamide gel electrophoresis (SDS-PAGE). After gel separation, the proteins were transferred to a PVDF membrane. The primary antibodies are diluted as the manufacturer’s recommendations: mouse anti-GRP78 (Santa Cruz), mouse nti-GADD153/C/EBP homologous protein (CHOP) (Santa Cruz) and mouse anti-β actin antibody (Sigma). The membranes were incubated for 1 h with either anti-mouse or anti-rabbit horseradish peroxidase IgG secondary antibodies (Sigma). Chemiluminescence detection using Western Lightning Chemiluminescence Reagent plus (Perkin Elmer) was performed. Membranes were exposed to imaging film (Kodak Bioflex Econo Scientific) and developed using a Kodak X-OMAT 1000A. The immunoreactive band was visualized by using the ECL detection reagent (Applygen Tech Inc, China). The densities were measured using a scanning densitometer coupled to scanning software (Image Quant; Molecular Dynamics, Amersham, Little Chalfont,UK). The expression of GRP78 and CHOP was evaluated and compared with the expression of β-actin. Adipose tissue cellular nuclear protein was extracted by nuclear protein extraction kit (Sigma). The expression of nuclear protein of NF-κB in adipose tissue was evaluated and compared with the expression of histone H(Santa Cruz). And the expression of nuclear protein of NF-κB in differentiated 3T3-L1 adipocytes was compared with the expression of nucleolin (BioVision).

### RNA isolation and real-time PCR

Total RNA was extracted from adipose tissue using Trizol reagent (Invitrogen) according to manufacturer’s instructions. RNA was reverse-transcribed using SuperScript III First-Strand Synthesis Supermix (Invitrogen). The cDNA samples were amplified in duplicate in 96-microtiter plates (Applied Biosystems). Each PCR reaction (20 μl of total volume) contained: 10 μl of SYBR Green PCR Master Mix (Applied Biosystems), 5 pmols of each primer, 1 μg of cDNA. Real-time PCR reactions were carried out in an ABI PRISM 7,500 real-time PCR apparatus. The thermal profile settings were 95 °C for 2 min, then 40 cycles at 95 °C for 10 s, 60 °C for 30 s and 70 °C for 45 s. The relative mRNA expression levels were normalized to expression of 28S rRNA.

### ELISA

Mouse TNF-α(R&D Systems, Inc) and resistin (R&D Systems, Inc) ELISA kits were used to assay secreted TNF-α and resistin from mouse serum and supernatant fluid from adipocytes. Assays were performed as per the manufacturer’s protocol. All samples were evaluated in triplicate. Intra-assay precision variability was <4% and 5.8% respectively.

### Histological analysis

Epididymal adipose tissue samples were fixed overnight in 10% neutral buffered formalin. Samples were then dehydrated with ethanol, embedded in paraffin, and sectioned (5 mm). H&E staining was performed using standard protocols. Analysis on cell size of adipocytes was conducted. At 10 magnifications, five representative images of each slide were captured. The number of adipocytes was counted, and the mean cell size of adipocytes was determined by dividing the total area of the image by the number of adipocytes.

### Lipid content measurement

(1) Using a spectrophotometer, serum total cholesterol (TC), triglyceride (TG), high-density lipoprotein cholesterol (HDL-C), low-density lipoprotein cholesterol (LDL-C) and glucose levels were determined by enzymatic colorimetric assays using commercial enzyme kits (Randox Laboratory, Crumlin, Northland, UK). (2) After collagenase digestion of epididymal adipose tissue, isolated adipocytes were harvested. Total cholesterol and free cholesterol were measured by cholesterol fluorometric assay kit (Sigma). (3) After blood collection, liver tissues were excised from mouse. To determine liver triglycerides, 20 mg of liver tissue was homogenized in a 200 μL solvent (chloroform:isopropanol:NP40 = 7:11:0.1). Centrifuged at 12,000 × *g* for 10 min, an aliquot of 100 μL was extracted and dried. The pellet was reconstituted with a buffer (1 M of potassium phosphate, pH = 7.4, 500 mM of sodium chloride, 50 mM of cholic acid), and water bath sonication was employed to dissolve the precipitate. A triglyceride colorimetric assay kit (Cayman, Ann Arbor, MI, USA) was used to analyze liver triglyceride contents.

### Statistical analysis

All data are presented as means ± SD of triplicate experiments. Comparisons among groups were performed by one-way ANOVA analysis with Bonferroni’s test for post hoc analysis. Statistical significance between two groups was determined using the Student’s t test. Differences were considered significant at a value of P < 0.05 for all tests.

## Results

### High-fat diet induced-obesity causes ER stress and chronic inflammation in adipose tissue

**Figure 1:**
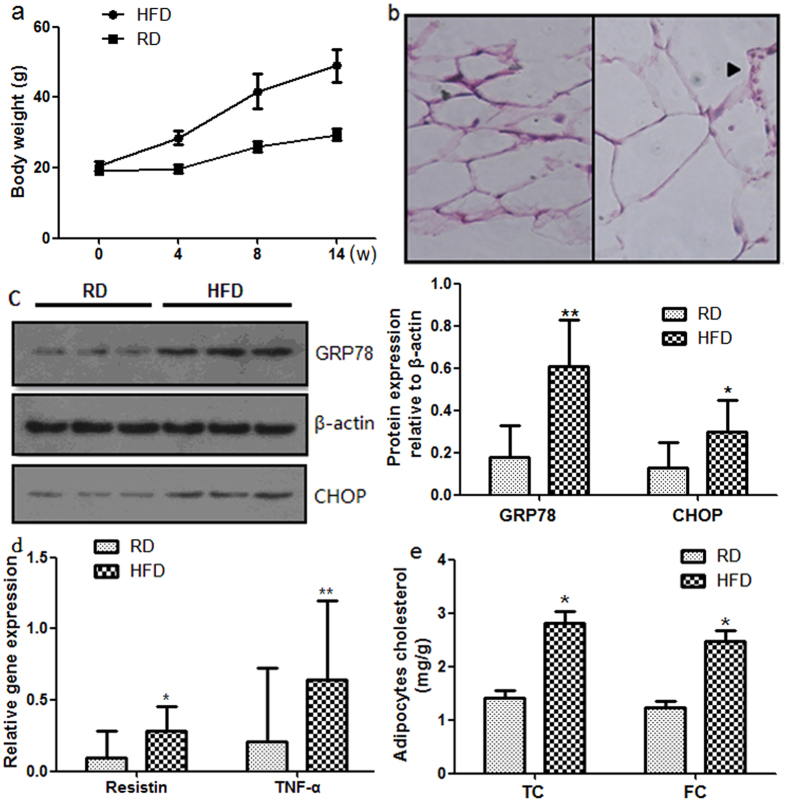
Chronic inflammation and ER stress in adipose tissue of high-fat diet induced-obesity. Mice were fed a regular diet (RD) or high-fat diet (HFD) for 14 weeks. (**a**) Body weights of HFD mice and RD mice. Mice fed a high-fat diet (n = 10) had higher weights compared with mice fed a regular diet (n = 10). (**b**) H&E staining of adipose tissue. Adipocytes hypertrophy and macrophage infiltration were observed in mice fed a high-fat diet for 14 weeks. (**c**) Protein expression levels of ER stress markers in adipose tissue. Protein expression levels of each ER stress marker were significantly up-regulated in mice fed a high-fat diet for 14 weeks. (**d**) Gene expression levels and blood protein levels of inflammatory cytokines in adipose tissue. mRNA expression levels of inflammatory cytokines were significantly up-regulated in mice fed a high-fat diet for 14 weeks. (**e**) The adipocytes TC and FC were elevated in high-fat diet mice. Data are presented as the means ± SD. *p < 0.05, **p < 0.01 (Compared with RD group).

To investigate ER stress *in vivo*, we studied diet-induced obesity mice, an important model for understanding the development of obesity. We fed male C57BL/6 mice a high-fat diet for 14 weeks. Body weight significantly increased in obese mice throughout the study period compared with control mice fed a regular diet (Fig. 1a).Furthermore, adipocytes from obese mice exhibited hypertrophy (Fig. 1b). Infiltration of macrophages and lymphocytes was also observed in adipose tissue (Fig. 1b). The expression levels of ER stress markers, glucose-regulated protein 78 (GRP78) and C/EBP homology protein (CHOP), were significantly up-regulated in adipose tissue of obese mice (Fig. 1c). We conducted RT-PCR analysis of TNF-α and resistin using mRNA isolated from epididymal adipose tissue of obese mouse. The expression levels of resistin and TNF-α were markedly elevated after 14 weeks of feeding (Fig. 1d). These findings provide evidence of chronic inflammation occurring in obese adipose tissue. We further determined the total cholesterol (TC) and free cholesterol (FC) in adipocytes form mouse epididymal adipose tissue. Both the adipocytes TC and FC were elevated in high-fat diet mice (Fig. 1e).Both of these parameters—blood triglyceride (1.09 ± 0.05 mmol/L versus 0.48 ± 0.14 mmol/L, P < 0.05) and serum glucose levels (13.5 ± 2.30 mmol/L versus 7.26 ± 1.25 mmol/L, P < 0.05)—were elevated in obese mice. Our findings suggested that ER stress, chronic inflammation and metabolic disorders occur in adipose tissue of obese mice. Taken together, these results indicate that the occurrence of adipose tissue inflammation in diet induced obesity may be intrigued by the activation of the ER stress signaling pathway in adipose tissue.

### Chemical chaperones modify the levels of inflammatory cytokines in mice fed a high-fat diet

**Figure 2:**
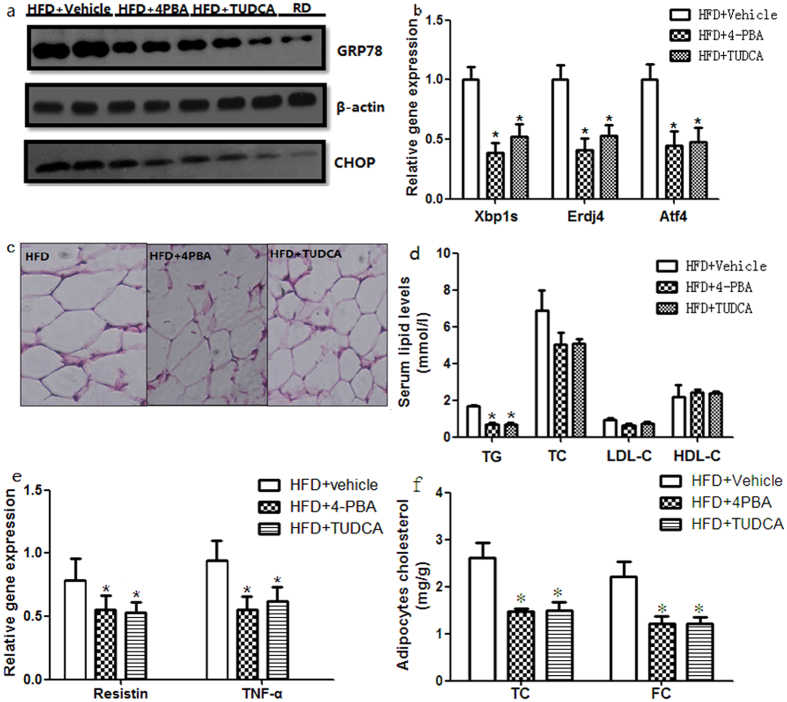
Chemical chaperones modify ER stress and inflammatory in mice fed a high-fat diet. Mice were fed a high-fat diet for 14 weeks and then treated with 4-PBA or TUDCA for 6 weeks at a dose of 1 g/kg/day. (**a**) Protein expression levels of ER stress markers in adipose tissue. Protein expression levels of ER stress markers were significantly depressed by treatment with 4-PBA or TUDCA. Values are means ± SD (n = 10). (**b**) Gene expression of UPR markers indicating the state of ER stress pathways in adipose tissue. The gene expression of Xbp1s, Erdj4 and Atf4 were also decreased by 4-PBA or TUDCA. Values are means ± SD (n = 10). (**c**) H&E staining of adipose tissue. Treatment with 4-PBA or TUDCA slightly decreased the size of the adipocytes. (**d**) Effects of 4-PBA or TUDCA on serum lipids and glucose concentrations of mice fed a high-fat diet. The blood glucose and triglyceride levels of obese mice treated with 4-PBA (n = 10) or TUDCA (n = 10) were significantly lower than those of vehicle-treated obese mice (n = 10). Serum TC and LDL-C had a slight but not significant decrease by the treatment of chemical chaperons. (**e**) Gene expression levels of inflammatory cytokines in adipose tissue. The mRNA expression levels of inflammatory cytokines were down-regulated by treatment with 4-PBA or TUDCA. (**f**) TC and FC in adipocytes form mouse epididymal adipose tissue after treatment of 4-PBA or TUDCA. Both the adipocytes TC and FC were decreased by the treatment of chemical chaperones (n = 10). Values are means ± SD. *p < 0.05 (compared with HFD + vehicle group).

To investigate the roles of ER stress in chronic inflammation, we examined the effects of ER stress on the expression of inflammatory cytokines in diet-induced obese mouse model by administering them to chemical chaperones that alleviate ER stress. Our previous study showed the chemical chaperones, 4-PBA and TUDCA, protected against experimental ER stress in adipocytes^15^. To investigate the action of chemical chaperones *in vivo*, we further studied diet-induced obesity mice. 4-PBA or TUDCA supplementation decreased ER stress markers GRP78 and CHOP expression in the adipose tissue in obese mouse, suggesting oral administration of chemical chaperones alleviates ER stress (Fig. 2a). Additionally, UPR markers indicating the state of ER stress pathways including the gene expression of X-box binding protein 1(Xbp1s), ER DnaJ homolog 4 (Erdj4) and transcription factor 4 (Atf4) are also decreased by the chemical chaperones (Fig. 2b). We then focused on downstream events of ER stress. In obese mice treated with 4-PBA or TUDCA, the adipocytes were smaller compared with those in vehicle-treated mice (Fig. 2c). Oral administration of 4-PBA or TUDCA to obese mice reduced ambient blood glucose to normoglycemic levels seen in the regular diet controls (8.25 ± 1.37 mmol/L, 8.75 ± 1.25 mmol/L versus 7.76 ± 2.30 mmol/L, p > 0.05). Serum lipid levels including cholesterol fractions in obese mouse were also modified by administration 4-PBA or TUDCA (Fig. 2d). We then performed RT-PCR to examine whether ER stress influences gene expression of inflammatory cytokines in adipose tissue. Treatment with 4-PBA or TUDCA significantly down-regulated the gene expression levels of resistin and TNF-α in adipose tissue of mice fed a high-fat diet (Fig. 2e). The decrease of serum levels of resistin and TNF-α was in consistent with the decrease of gene expression levels (P < 0.05). We further determined the total TC and FC in adipocytes form mouse epididymal adipose tissue after treatment of 4-PBA or TUDCA. Both the adipocytes TC and FC were decreased by the treatment of chemical chaperones (Fig. 2f). Triglyceride in liver tissue was also decreased (650.25 ± 28.77 μg/g, 644.37 ± 26.25 μg/g versus 872.36 ± 33.34 μg/g, P < 0.05). These results suggest the reversal of hypertriglyceridemia, blood glucose, and the decrease of inflammatory cytokines as well as adipose tissue and liver lipids are related to modification of ER stress in obese mice and chemical chaperones prevent the inflammatory response in adipose tissue.

### Chemical chaperones suppress NF-κB activity in adipose tissue of mice fed a high-fat diet

**Figure 3:**
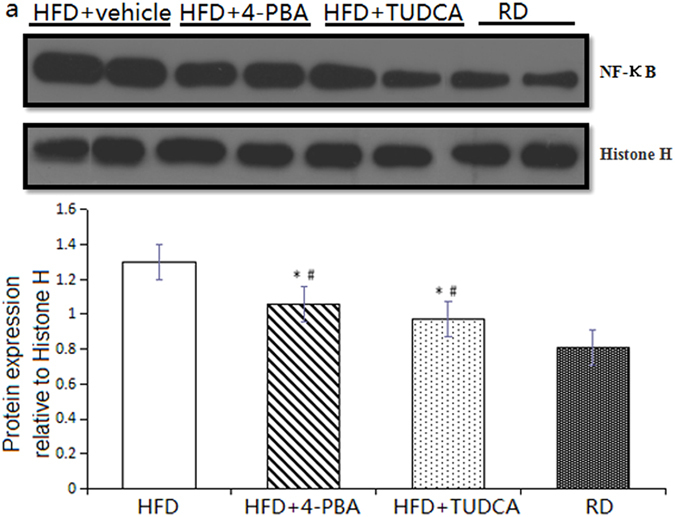
Chemical chaperones suppress NF-κB activity in adipose tissue of mice fed a high-fat diet. (**a**) Nucleus expression levels of NF-κB in adipose tissue were down-regulated by treatment with 4-PBA or TUDCA. Values are means ± SD (n = 10) *p < 0.05 (compared with HFD + vehicle group), ^#^p < 0.05 (compared with RD group).

Nuclear factor-κB (NF-κB) plays an important role in adipose tissue inflammation in human and experimental models of obesity and is the main transcription factor in ER stress-mediated inflammation. We then performed western-blot to investigate whether NF-κB pathway participates in ER stress induced adipose tissue inflammation. C57BL/6 mice fed with a high fat diet supplemented with 4-PBA or TUDCA showed a significant reduction in nuclear protein NF-κB expression in adipose tissue compared with those in vehicle-treated mice (Fig. 3a), accompany with modification of adipose tissue inflammation.

### IKK/NF-κB may be involved in the signal transduction of adipokine expression regulating by ER stress

**Figure 4:**
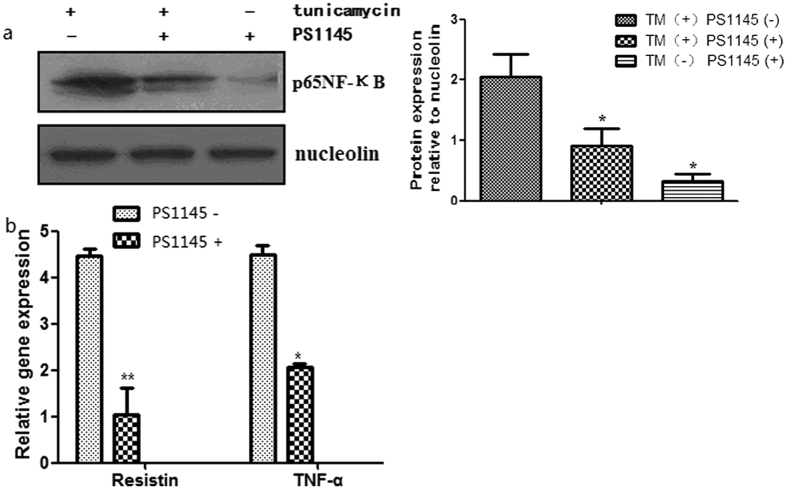
IKK/NF-κB pathway in the signal transduction of adipokine expression regulating by ER stress. 3T3-L1 preadipocytes were cultured and induced to differentiate into mature adipocytes. ER stress was triggered by TM, then PS1145 was added to inhibit NF-κB pathway. (**a**) Nucleus expression levels of NF-κB in adipocytes were down-regulated by treatment with PS1145. (**b**) Gene expression levels of inflammatory cytokines in adipose tissue. The mRNA expression levels of resistin and TNF-α were down-regulated by treatment with PS1145. Values are means ± SD. *p < 0.05, **p < 0.01 (compared with control group).

Based on our observations *in vivo*, we hypothesized that IKK/NF-κB may participate in the signal transduction in the adipokine release disorder of adipocyte caused by intracellular ER stress. To investigate whether IKK/NF-κB pathway was involved in the ER stress induced adipocytes inflammation, *in vitro* experiments using cultured adipocytes were performed. We stimulated cultured adipocytes with 0.6 mg/ml tunicamycin, which causes ER stress by inhibiting N-linked glycosylation (Fig. 4a). Then NF-κB inhibitor PS1145 was added. This treatment significantly depressed mRNA expression of resistin and TNF-α in 3T3-L1 adipocytes (Fig. 4b). After the intervention with 0.6 mg/ml TM and then added with NF-κB inhibitor PS1145, resistin (23.45 ± 2.33 pg/ml versus 11.74 ± 1.75 pg/ml, P < 0.05) and TNF-α (375.25 ± 12.83 pg/ml versus 208.21 ± 10.30 pg/ml, P < 0.05) levels in the supernatant fluid from adipocytes were also decreased. Taken together, these findings indicate that ER stress may regulate the expression of adipokines at least partly via stimulating NF-κB activity.

## Discussion

Studies in the past decade have demonstrated that obesity is associated with chronic inflammation and established a link between inflammatory responses and development of obesity related metabolic disorders. The inflammatory role of adipocytes is related to its expansion, hyperplasia and hypertrophy. However, the molecular mechanisms and signals that trigger chronic inflammation in obesity are not well understood. In the present study, we showed activation of the inflammatory response and metabolic disorders in adipose tissue of mice fed high-fat diet. These pathophysiologic events were alleviated by chemical chaperones that can suppress ER stress. A variety of cellular stresses like ER stress, mitochondrial dysfunction, oxidative stress are involved in the pathogenesis of obesity^16^,^17^. But current pharmacotherapeutic options for managing cellular stresses and modifying obesity-related disorders remain limited and ineffective. The chemical chaperones we studied, 4-PBA and TUDCA, have been approved by the US. Food and Drug Administration for clinical use in diseases such as cholelithiasis and cholestatic liver disease^18^. Several recent studies have indicated that, in obese humans, chemical chaperones directly decrease the lipid content of adipocytes and reduce body mass^19^ and improved systemic insulin sensitivity in obese mouse model^20^. Our findings showed that alleviation of ER stress modified serum dyslipidemia, serum glucose and secretion profile of adipokines of diet-induced obesity mice. These results may warrant clinical investigation for chemical chaperones, 4-PBA and TUDCA in particular, as treatments for metabolic disorder in obesity.

Obesity is associated with disorders in lipid metabolism, typically hypertriglyceridemia and low-high density lipoprotein-cholesterol levels. The present study revealed the possibility that inhibition of ER stress may be an effective approach to reduce serum lipids, mainly serum TG, intriguing the interest to explore the potential mechanism. Fat cells are the largest lipid pool. A decrease in cell sizes and triglyceride content of adipocytes from obese mice treated with TUDCA and 4-PBA was detected, indicating that lipids deposit in adipose tissue decreased. We further revealed that triglyceride of liver tissue form diet-induced obese mice was also decreased by the treatment of chemical chaperones. Consistent with this, Ozcan *et al.* showed TUDCA and 4-PBA treatment in ob/ob mice resulted in resolution of obesity-induced TG content accumulation in liver^20^. These results suggested that the decrease of serum TG in obese by chemical chaperons may partly due to accelerate TG metabolism in liver, which worth further exploration.

The decisive origins of ER stress in obesity, particularly in adipose tissue is unknown, but several possibilities exist, such as free fatty acid-mediated reactive oxygen species (ROS) generation, nutrient overload and local glucose deprivation. In our previous study, we showed that feeding adipocytes with oxidized low density lipoprotein (ox-LDL) increased intracellular FC and caused widespread ER stress, suggesting the possible role of excess FC in obesity-induced ER stress in adipocytes. Consistent with our result, it has been reported that intracellular accumulation of excess free cholesterol induces macrophages secretion dysfunction via ER-stress pathway^21^.Adipose tissue is the body’s largest pool of free cholesterol. Many studies highlighted cholesterol imbalance in enlarged adipocytes in the obese state. In fact, excess free cholesterol is deleterious to cells^22^. Since ER has very low cholesterol content, the accumulation of free cholesterol in this cellular organelle may induce membrane dysfunction and subsequent ER stress. Therefore it could be presumed that cholesterol load may be increased in adipocytes through endocytosis and degradation of ox-LDL, which subsequently result in the activation of ER stress. Consistent with this, we found the down-regulation of ER stress markers is in accordance with the decrease of FC in adipocytes from adipose tissue of obese mouse. Though need further study, this provides another mechanism for the induction of adipocytes ER stress by ox-LDL in addition to oxidative stress.

NF-κB plays an important role in adipose tissue inflammation in human and experimental models of metabolic disease^23^,^24^ and is the main transcription factor in ER stress-mediated inflammation^25^,^26^. In this study we found that NF-κB was activated in adipose tissue from high fat diet induced obese mice as well as in hypertrophic 3T3-L1 adipocytes. Adipokines are important factors connecting adipose tissue inflammation with systemic metabolic disorder. Therefore, we suggest that NF-κB activation could be a key process in ER stress- mediated cytokines factors expression and release, such as TNF-α and resistin, among others. Three major UPR signaling pathways mediated by PERK, IRE1, and ATF6 are activated by ER stress^27^. *In vitro* studies showed NF-κB pathway was activated by PERK and ATF6 transduction^28^. Further efforts should be made to explore the signaling pathways in adipocytes in obesity.

To conclude, the results of this study demonstrate that ER stress is activated in adipose tissue in high fat diet-induced obese mice and mediated downstream inflammatory responses. The chemical chaperones, 4-PBA and TUDCA, modified adipose tissue inflammation and systemic metabolic disorders in obesity via restoring ER stress. The accumulation of FC in adipocytes of obesity may one of the trigger factors that induce intracellular ER stress. Adipokine expression associated with obesity and obesity-related metabolic diseases. Although various inflammation pathways have been identified as regulating ER stress and its stress responses in adipose tissue, we propose that NF-κB is a vital regulator in adipokines expressed and secreted from adipocytes.

## Additional Information

**How to cite this article**: Chen, Y. *et al.* Chemical chaperones reduce ER stress and adipose tissue inflammation in high fat diet-induced mouse model of obesity. *Sci. Rep.* **6**, 27486; doi: 10.1038/srep27486 (2016).
